# Synchrotron radiation-based phase-contrast microtomography of human dental calculus allows nondestructive analysis of inclusions: implications for archeological samples

**DOI:** 10.1117/1.JMI.9.3.031505

**Published:** 2022-03-16

**Authors:** Robert C. Power, Amanda G. Henry, Julian Moosmann, Felix Beckmann, Heiko Temming, Anthony Roberts, Adeline Le Cabec

**Affiliations:** a Ludwig-Maximilians-University Munich, Institute for Pre- and Protohistoric Archaeology and Archaeology of the Roman Provinces, Munich, Germany; b Max Planck Institute for Evolutionary Anthropology, Department of Human Evolution, Leipzig, Germany; c Leiden University, Faculty of Archaeology, Leiden, The Netherlands; d Helmholtz-Zentrum Hereon GmbH, Geesthacht, Germany; e Cork University Dental School and Hospital, University College Cork, Ireland; f Univ. Bordeaux, CNRS, MCC, PACEA, UMR 5199, Pessac, France

**Keywords:** archeology, micro-computed tomography, archeobotany, dental pathology, diet, microremains

## Abstract

**Purpose:**

Dental calculus forms on teeth during the life of an individual and its investigation can yield information about diet, health status, and environmental pollution. Currently, the analytical techniques used to visualize the internal structure of human dental calculus and entrapped inclusions are limited and require destructive sampling, which cannot always be justified.

**Approach:**

We used propagation phase-contrast synchrotron radiation micro-computed tomography (PPC-SR-μCT) to non-destructively examine the internal organization of dental calculus, including its microstructure and entrapped inclusions, on both modern and archeological samples.

**Results:**

The virtual histological exploration of the samples shows that PPC-SR-μCT is a powerful approach to visualize the internal organization of dental calculus. We identified several important features, including previously undetected negative imprints of enamel and dentine growth markers (perikymata and periradicular bands, respectively), the non-contiguous structure of calculus layers with multiple voids, and entrapped plant remains.

**Conclusions:**

PPC-SR-μCT is an effective technique to explore dental calculus structural organization, and is especially powerful for enabling the identification of inclusions. The non-destructive nature of synchrotron tomography helps protect samples for future research. However, the irregular layers and frequent voids reveal a high heterogeneity and variability within calculus, with implications for research focusing on inclusions.

## 1 Introduction

Dental calculus, also known as tartar, is increasingly targeted in archeological studies for its ability to preserve objects and residues that can provide information about past human diets and behaviors.^1^^–^^4^ Calculus is formed when oral plaque, a bacterial pellicle, is mineralized through the precipitation of salivary calcium phosphates.^5^^–^^7^ The mineralized surface of the forming calculus is re-colonized by an oral bacterial biofilm, which is then mineralized at a later point. This process creates a layered structure, and objects that were in the oral cavity, such as microscopic botanical remains, mineral particles, and a variety of biomolecules, such as deoxyribonucleic acid (DNA), proteins, and lipids, become encased and protected within the mineral structure of the dental calculus.^2^^,^^8^^–^^12^ A variety of archeological studies have isolated and identified these inclusions, to record the consumption of particular food items,^1^^,^^4^ to describe oral bacterial communities,^1^^,^^11^ and to reconstruct human non-dietary behavior such as paint preparation^2^ and use of burial goods.^1^^,^^2^^,^^5^^,^^13^^–^^15^

Despite the success of these methods, several questions about dental calculus as a record of past diets and behaviors remain unanswered. First, though it is widely understood how dental calculus formation occurs and how salivary calcium phosphates inhibit demineralization of enamel and dental decay,^6^ the mechanisms by which objects become entrapped and preserved are unclear. Second, most methods to extract and analyze the internal structure or the inclusions are destructive. In most analyses, the calculus is either entirely broken down, chemically or mechanically, to isolate the inclusions, or it is cut following histological or thin-sectioning methods.^1^^,^^5^ The former methods may damage fragile inclusions, particularly calcium-based inclusions due to the use of acidic or chelating chemicals, while the latter results in the loss of most of the sample during the preparation of the section. Therefore, valuable data about the formation process of the calculus and the relative position of certain inclusions in three-dimensional (3D) space is lost. Furthermore, destructive sampling methods are not always permissible on archeological material (see Ref. 16 for a discussion).

Several researchers have explored non-destructive methods to visualize the microscopic structures and internal features of archeological and fossil materials. Scanning electron microscopy has been used to examine the internal structures and inclusions of dental calculus, but only on already-broken surfaces or histological thin sections.^1^^,^^13^^,^^17^^–^^19^ Other methods, such as conventional micro-computed tomography (μCT), are better suited for visualizing internal structures in undamaged specimens, and have been successfully used on other kinds of archeological specimens such as pottery and charred plant seeds.^20^ However, using conventional μCT, the intensity contrast of inclusions and structures of interest in calculus is typically too small to visualize them. High-resolution analyses of internal features are possible with propagation phase-contrast synchrotron radiation μCT (PPC-SR-μCT). Although researchers have used this method to explore the microstructure in fossil teeth to understand the dental development of extinct taxa,^21^^–^^28^ to date, no studies have used PPC-SR-μCT to visualize the 3D structure of dental calculus.

In this study, we report preliminary results of the analyses of two dental calculus samples scanned using PPC-SR-μCT at the beamline P07 operated by Hereon at the PETRA III storage ring (DESY, Hamburg, Germany). This pilot study aims to: (1) test whether PPC-SR-μCT enables the non-destructive visualization the inner microstructure of dental calculus in both isolated chunks and *in situ* samples (on teeth); (2) compare the visibility of the fine calculus inner structure between PPC-SR-μCT and conventional μCT; (3) see whether inclusions can be visualized and identified; and (4) explore whether the fine microstructure of the calculus deposits can be observed and the formation process be discussed. Because this pilot study also aims at introducing this approach using x-ray imaging to archeologists, we provide detailed explanations and technical parameters.

## 2 Materials

**Fig. 1:**
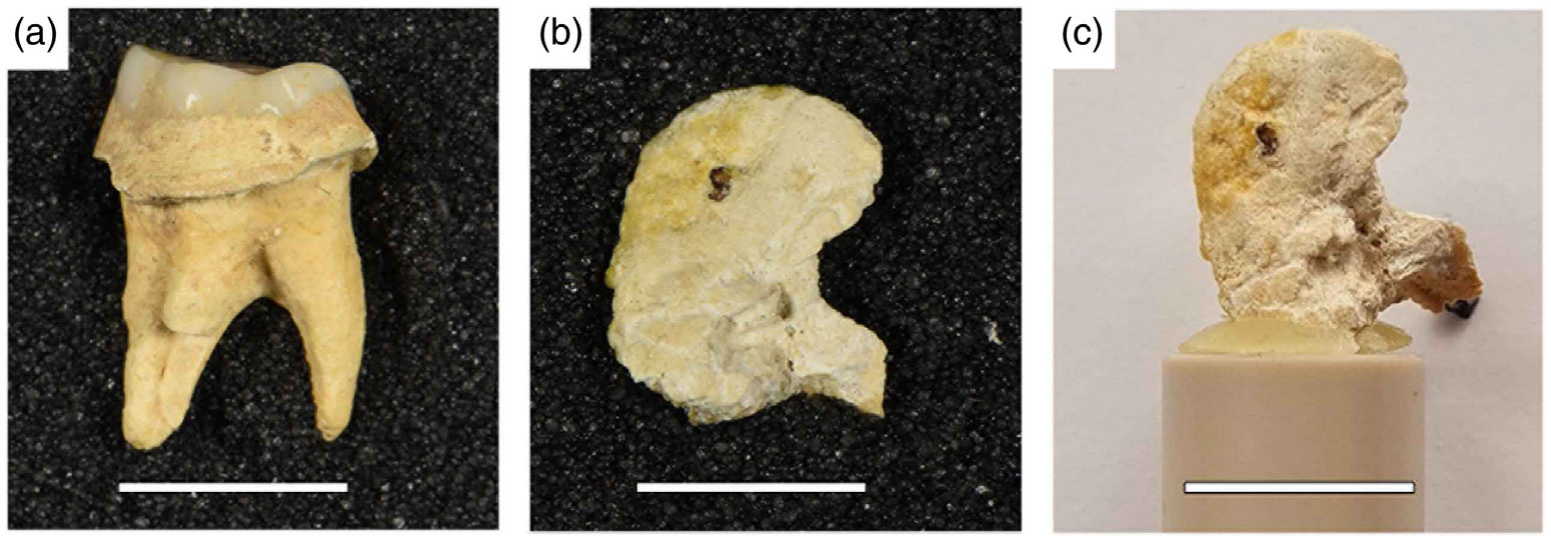
(a) Macrophotograph of the archeological dental calculus sample on the tooth (FK01). The tooth is from Cist 2, Fourknocks II (Ireland); (b) macrophotograph of the modern isolated dental calculus sample from a clinical setting (CM03); and (c) CM03 positioned and fixed on the sample holder with beeswax. The samples were mounted above the sample holder to minimize attenuation during scanning. The scale bar applies to all subfigures and is 1 cm.

We examined two samples of dental calculus: one contemporary (CM03) and one archeological (FK01) (Fig. 1). CM03 was removed from a 36-year-old male smoker as part of their clinical management. It was collected from the supra-gingival buccal surface of the mandibular left second premolar and mandibular left first molar. This calculus deposit had formed over the course of 1 year and was removed atraumatically from the patient, avoiding any blood contamination of the sample. Tobacco smoking is known to have a strong impact on dental calculus deposition in the clinical literature,^29^^,^^30^ which could account for the non-negligible amount of calculus formed on these teeth within 1 year. The ethical approval to collect and study this material was granted by the Research Ethics Committee of the Cork Teaching Hospitals, Ireland (2018 meeting).

FK01 formed on the upper right second molar of a roughly 25-year-old female individual recovered from a Chalcolithic burial from Fourknocks II in Ireland.^31^ This individual was buried in a secondary cist (Cist 2) inserted into the covering mound of a Neolithic Passage Tomb. The burial has been directly dated to 2450–2030 cal BC (UBA-29672 3780±53).^32^ The calculus was not removed from the tooth before scanning; instead, the whole tooth was mounted on the sample holder. It is not possible to determine the length of time over which this calculus deposit was formed but given the absence of modern dental hygiene practices in prehistory, it is likely that this deposit represents years to decades of the individual’s life.

## 3 Methods

### 3.1 Micro-Tomographic Data Acquisition and Reconstruction

The dental calculus chunk (CM03) and the tooth (FK01) were individually mounted onto the sample holder (plastic pin) using small slivers of beeswax [Fig. 1(c)]. The sample holder was then placed on the sample stage for centering the sample into the field of view (FOV) using radiographic mode.

#### 3.1.1 Propagation phase-contrast synchrotron radiation micro-computed tomography

Experiments were performed at the P07 High Energy Material Science Beamline (HEMS),^33^ which is operated by Helmholtz–Zentrum Hereon at PETRA III at Deutsches Elektronen-Synchrotron (DESY) in Hamburg, Germany, both members of the Helmholtz Association HGF. The storage ring was operated in 480-bunch using top-up filling mode with a current of 120±0.5  mA. The primary x-ray beam was monochromatized to 64 keV using a double crystal monochromator [bent Laue, Si(111)]. For this experiment, an indirect detector system was used comprised of a 20 MPixel CMOS (CMOSIS CMV20000 sensor) camera,^34^ developed by HEREON and the Karlsruhe Institute of Technology, a 100-μm thick ${\mathrm{CdWO}}_{4}$ scintillator and a 5× microscope optics. An HDF5 log file was recorded for each scan to keep track of the parameters. Detailed parameters are given in Table 1.

**Table 1:** Technical parameters used to scan both calculus samples (CM03 and FK01) by PPC-SR-μCT on the beamline P07 at DESY.

	FK01	CM03
Energy (keV)	64	64
Ring current (mA)	120	120
Number of projections	10001	10001
Exposure time (ms)	75	200
Propagation distance (sample to detector distance in mm)	300	300
Number of scans in height	1	6 (δz=2.15 mm, with 606 pixels overlap)
Number of lateral scans	1	2
Dark images (at the beginning of each acquisition)	20	20
Reference images (at the beginning and every 1000 projections over 360 deg)	40 (440 in total)	20 (420 in total)
Camera		
Aperture opening	1.00	0.67
FOV full chip (X×Y in mm)	6.52×4.89	6.51×4.88
FOV region of interest chip	6.52×2.80	6.51×2.92
FOV stitched	—	12.51×13.68
Magnification	5.033	5.032
Effective pixel size (μm)	1.273	1.272

The PPC-SR-μCT data were reconstructed using a routine,^35^^,^^36^ implemented in MATLAB [version: 9.4.0.813654 (R2018a)] for the single FOV scans. Projections of the samples scanned with several lateral or vertical FOVs were processed and stitched using interactive data language (IDL, Harris Geospatial Solutions, Inc.) applying a binning of 2, resulting in a pixel size of 2.55  μm. The ASTRA toolbox was then used for tomographic backprojection.^37^^,^^38^ The datasets were then cropped to remove the part of the volume only containing air and the pad of wax. To do so, a maximum intensity projection horizontally was used to find the outline of the sample in Fiji.^39^ This enabled us to save up to 50% (in the case of CM03) of the size of the original volume, and thus facilitate later data handling. The reconstructed PPC-SR-μCT data were finally converted from 32-bit into 16-bit tiffs using Fiji,^39^ with an adjustment of the contrast on a representative slice and a slight increase of the dynamic range.

#### 3.1.2 Conventional micro-computed tomography

Both specimens were scanned at 5.86  μm on the diondo d3 (diondo GmbH, Hattingen, Germany) industrial μCT scanner of the Department of Human Evolution, at the Max Planck Institute for Evolutionary Anthropology (MPI-EVA, Leipzig, Germany). The raw data were reconstructed in Siemens Cera 3.0.2., which yielded stacks of 8-bit tiff files. Detailed parameters for the acquisition and reconstruction are given in Table 2.

**Table 2:** Acquisition and reconstruction parameters for the conventional μCT scans of both calculus samples (CM03 and FK01) at MPI-EVA.

	FK01	CM03
Acquisition (diondo d3 industrial μCT scanner)		
Voltage (kV)	120	120
Current (μA)	50	50
Number of projections	2970	2970
Frame average	2	2
Exposure time (ms)	1500	750
X-ray source to sample distance (mm)	32	32
X-ray source to detector distance (mm)	758.807	758.979
X-ray metallic filter	0.5-mm Brass	0.25-mm Brass
Number of scans in height (∼15.6% overlap at top and bottom)	3	3
Number of lateral scans	1	1
Binning during scan	1×1	1×1
Scan angle	360 deg	360 deg
Scan time	14 h 51 min	7 h 25 min
Reconstruction (Siemens Cera 3.0.2.)		
Data type	8 bit tiff	8 bit tiff
Ring artifact correction	2	1
Effective pixel size (μm)	5.8618	5.8605

### 3.2 Virtual Histology

The tiff stack of PPC-SR-μCT data was opened and further processed in VG Studio MAX 3.5.0 (64 bit, Volume Graphics GmbH, Heidelberg, Germany). Virtual histology techniques were employed to explore the volume. The surface features were visualized using Phong’s volume-rendering algorithm combined with colored lights.^26^ Therefore, all 3D models shown in the figures show the samples with false colors, chosen by the user. Virtual two-dimensional (2D) sections of various thickness^40^ were recorded to best reveal the inner structures of the samples.

## 4 Results

### 4.1 Non-Destructive Synchrotron Phase-Contrast Imaging

Both the isolated chunk of dental calculus and the calculus remaining on the tooth were successfully imaged with no visible mechanical damage or color change. Mounting these samples using beeswax proved a successful means to stabilize the samples while retaining the ability to remove the calculus or tooth after scanning to perform further analyses or to return the sample to the museum. The scans revealed a variety of information about the formation of calculus and inclusions. We note that the image quality is better in CM03 than in FK01: the spatial resolution and the signal-to-noise ratio are much improved since CM03 was scanned with a smaller aperture (0.67 versus 1.00) and a higher exposure time (200 versus 75 ms; see Table 1).

### 4.2 Calculus Microstructures

**Fig. 2:**
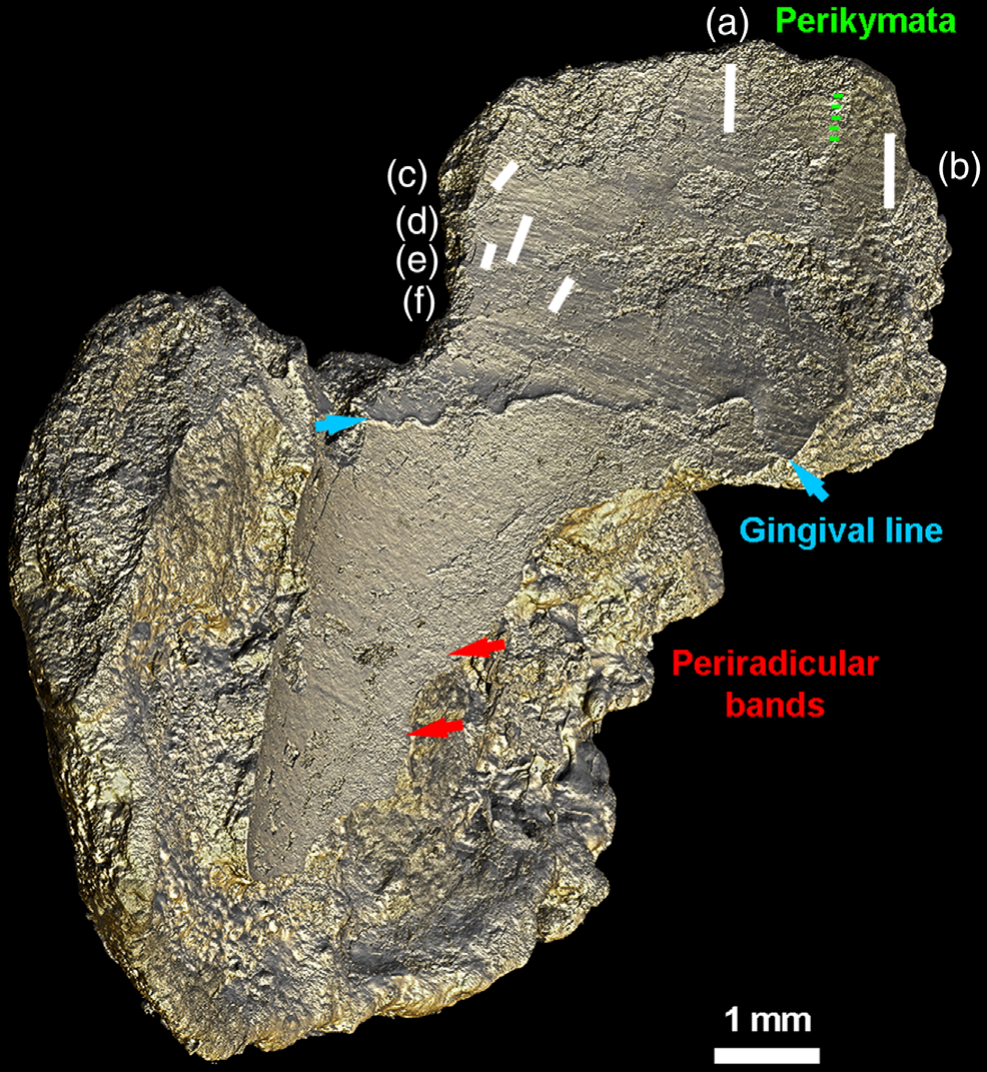
Lingual view of the 3D rendering (Phong algorithm, colored lights; see Ref. 26 for details) of the CM03 modern calculus specimen. Part of the calculus was in direct contact with either the mandibular second premolar or first permanent molar. This left negative imprints of the growth increments on the surface of the enamel crown (termed “perikymata”; green lines), of the gum line (blue arrows), and of dentine accentuated incremental features on the tooth root (called periradicular bands; red arrows). Average spacing between structures identified as perikymata are consistent with published values^41^: (a) 93, (b) 103, (c) 63, (d) 53, (e) 42, and (f) 44  μm.

Based on the exploration of the high precision 3D models of the surface of the calculus, we could identify interesting and relevant microfeatures. First, in the isolated calculus chunk (CM03), it was straightforward to identify the surface of the dental calculus that once adhered to the tooth surface by its smooth profile. This interior surface also retained negative impressions of regular and periodic incremental markers (Fig. 2), much like a high-quality dental cast. In the middle, at the junction with the free rim of the specimen, a wavy line of substantial thickness marks the gingival line (Fig. 2). Above this, on the surface of the calculus in direct contact with the enamel crown, periodic grooves show an average spacing ranging from 38 to 65.4  μm in the most cervical part, and from 92 to 104  μm in what corresponds to the middle third of the crown enamel (Fig. 2). These were identified as negative impressions of perikymata (i.e., enamel growth markers), in agreement with the wide ranges of perikymata spacing values provided for ancient human teeth.^41^ Below the impression of the gingival line, fainter curved lines represent the impression of periradicular bands, which are root dentine growth markers. Since these bands are often covered by a thin layer of cementum, it is not reliable to measure their spacing, due to the uncertainty of clearly identifying two consecutive periradicular lines, and to the higher probability of seeing only accentuated lines.^26^^,^^42^^,^^43^ The surface in contact with the oral environment could be readily distinguished by its complex texture.

**Fig. 3:**
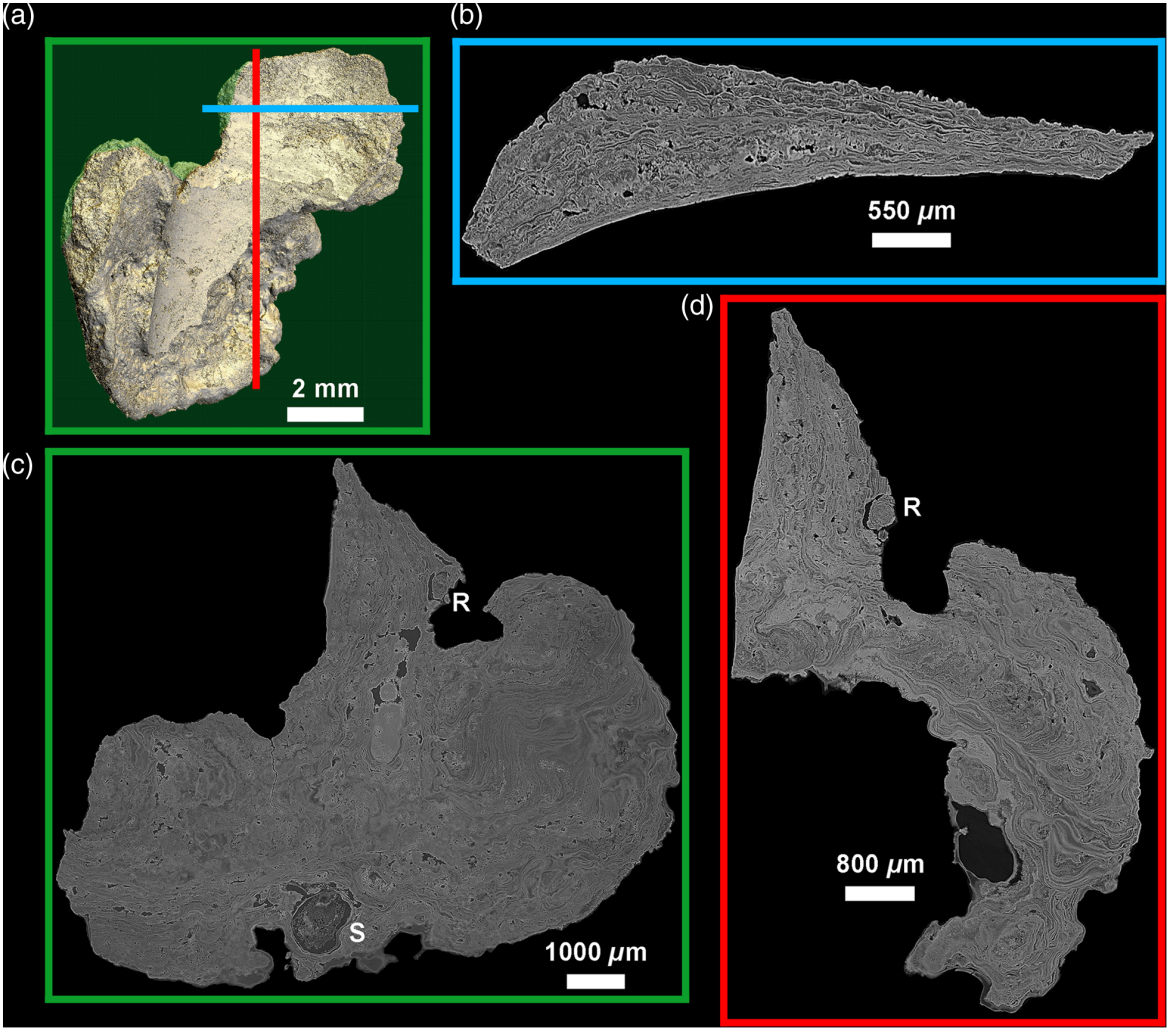
3D model of the CM03 specimen [(a) Phong algorithm, colored lights] showing the location of the 24  μm-thick virtual 2D sections in the three orthogonal planes of space (b) blue; (c) green; (d) red. This reveals the extensive incremental structure of this modern calculus involving fine and scalloped growth layers of various thickness, likely resulting from differences in deposition rates, with convoluted patterning, not parallel to the tooth surface, nor to the calculus surface. Some broader layers show a slightly higher density and a more homogeneous structure (light gray). R: vegetal rod inclusion, shown in Fig. 8. S: poppy seed inclusion, shown in Fig. 7.

**Fig. 4:**
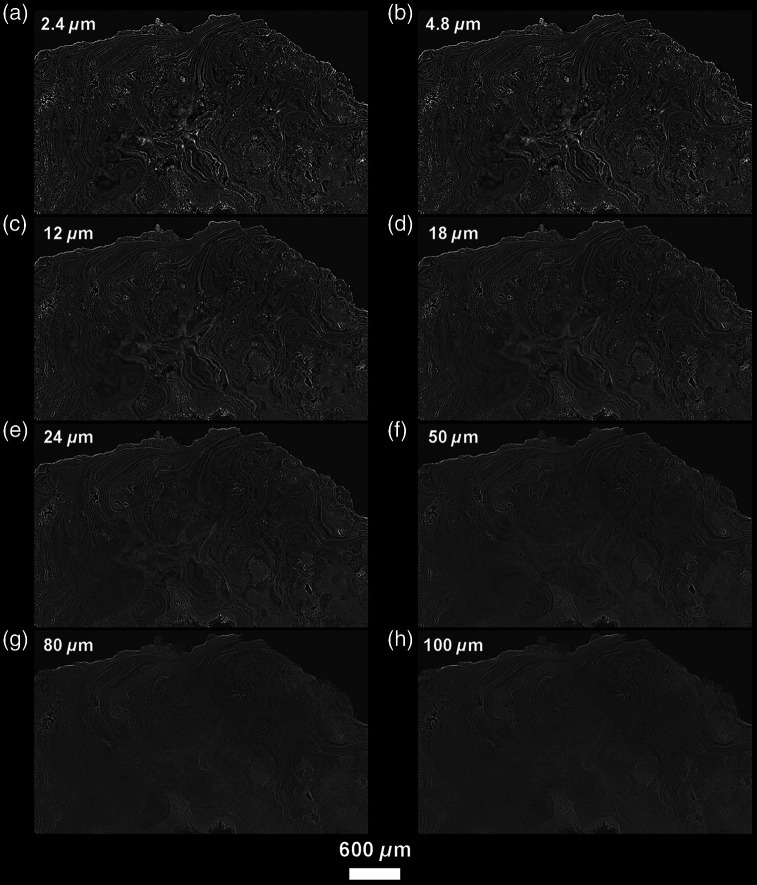
Variation in virtual slice thickness from a single reconstructed PPC-SR-μCT slice (with a binning of 2: 2.4-μm thick) to the averaging of multiple slices to reach from 4.8 to 100  μm in thickness (a)–(h), in the modern calculus sample CM03. It is noteworthy that there is a considerable loss of details for slice thicknesses comparable with values used in classical histology (from 24 to 100  μm). Slices that are 12- to 18-μm thick seem to best preserve details with an enhanced visibility due to the noise reduction resulting from the averaging of multiple slices (see Ref. 40 for details).

**Fig. 5:**
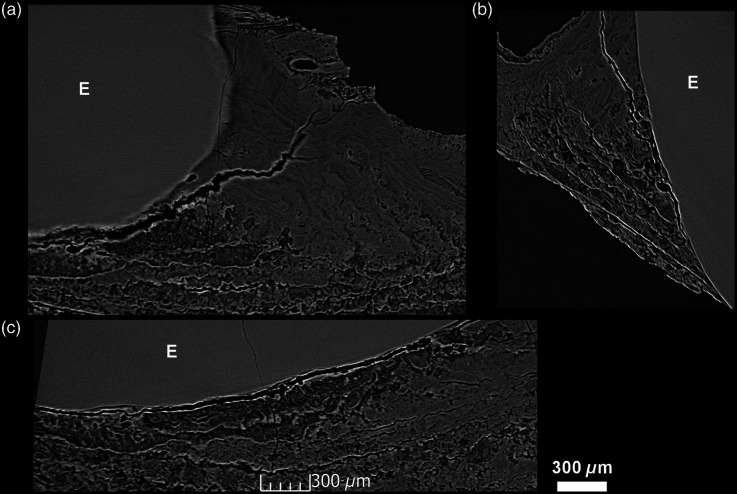
Archeological specimen FK01 shows a layered structure similar to that observed in CM03 (Fig. 3). On the 12-μm-thick virtual slices (a)–(c), taphonomic processes may have induced local and partial demineralization of the layers. They nevertheless preserve their convoluted shapes that diverge from both the tooth and calculus surface. E: enamel.

**Fig. 6:**
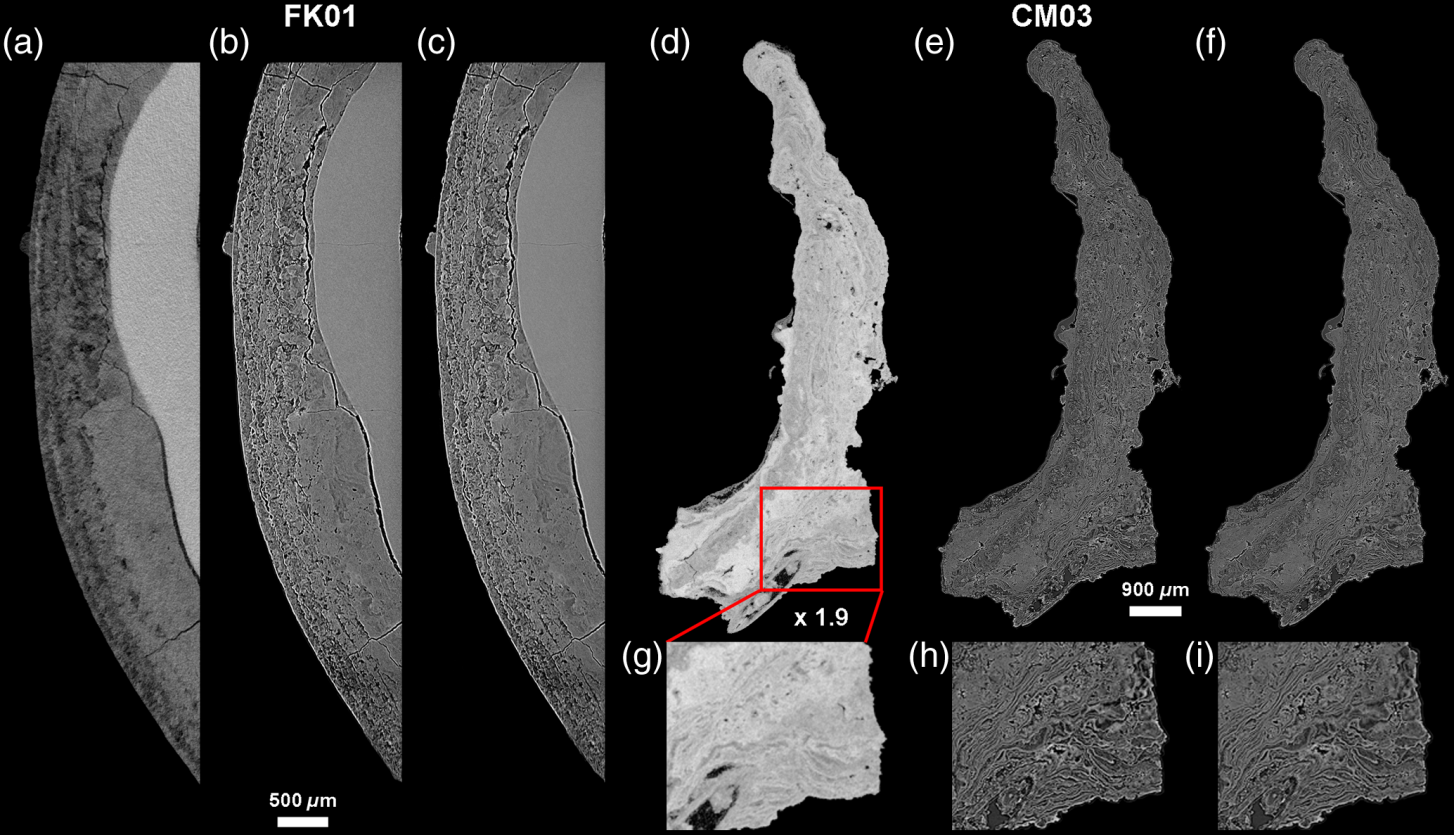
Comparisons of conventional μCT and PPC-SR-μCT results. (a)–(c) are the archeological specimen FK01, and (d)–(i) are the modern clinical sample CM03. (a), (d), and (g) were captured using conventional μCT with a 5.86  μm slice thickness, while (b), (e), and (h) show PPC-SR-μCT scans at 2.5  μm, and (c), (f), and (i) are virtual sections averaging several slices to reach a thickness of 5.86  μm. The difference between the two types of PPC-SR-μCT is negligible, while the fine microstructure of the dental calculus, especially the layers, are much easier to identify than on the conventional μCT data.

Using virtual histology techniques, and especially 2D thick-slabs (i.e., the averaging of multiple contiguous 2D slices to reach a desired slab thickness), the scans also allow us to visualize the complex interior structures of the calculus that are related to how it formed on the tooth. Both samples show multiple levels of layering, which exhibit as alternating heterogeneous thick layers with increments of varying density. Unexpectedly, many of these layers are not parallel to the calculus surface, the tooth surface, or other layers. Instead, they occur as highly convoluted, marbled forms (Figs. 3–5). Figure 3 shows the two major types of layers that can be distinguished in dental calculus: the very thin and contrasting layers of a few microns thickness, and broader, more homogeneous, light gray layers reaching several tens of microns in thickness. Some of the layers curve around inclusions. Notably, the complexity of this layering is only apparent at the highest resolutions, which is only possible with PPC-SR-μCT. Figure 6 shows this point by comparing the virtual sections of both the CM03 modern and FK01 archeological specimens scanned using a conventional x-ray lab source and a synchrotron x-ray source with phase-contrast imaging. Although the presence of the layers is indicated in the conventional μCT scans, only with phase contrast imaging is it possible to visualize their fine microstructure and borders. Figure 4 shows how these layers become optimally observable when individual PPC-SR-μCT slices are averaged, although within limits. Indeed, when the virtual thickness is comparable with those of histological sections of calculus, the layers and other microfeatures appear blurred (Fig. 4).

The modern calculus sample (CM03) also retains voids that seemingly follow intra-layer spaces and are of highly variable sizes, from a few tens to several hundreds of microns in diameter. The smaller voids often have a very elongated shape and scalloped outline, and lie roughly parallel to the layers of calculus, while the larger ones have irregular outlines, and possibly are the result of the decay or dissolution of organic inclusions. Voids also occur in the archeological sample FK01, but these are much larger (up to >1  mm), much more irregular, and often communicate with each other and open to the exterior.

In both the clinical and archeological samples, cracks are often seen running parallel to the layers of calculus, likely because these are areas of mechanical weakness. Cracks that run perpendicular to the calculus layers and connect the tooth surface (crown enamel or root dentine) to the exterior also occur. Although FK01 appears as an extremely well-preserved sample of calculus on its exterior (Fig. 1), it displays far more cracks and discontinuities inside than does CM03 (Fig. 5).

### 4.3 Inclusions

**Fig. 7:**
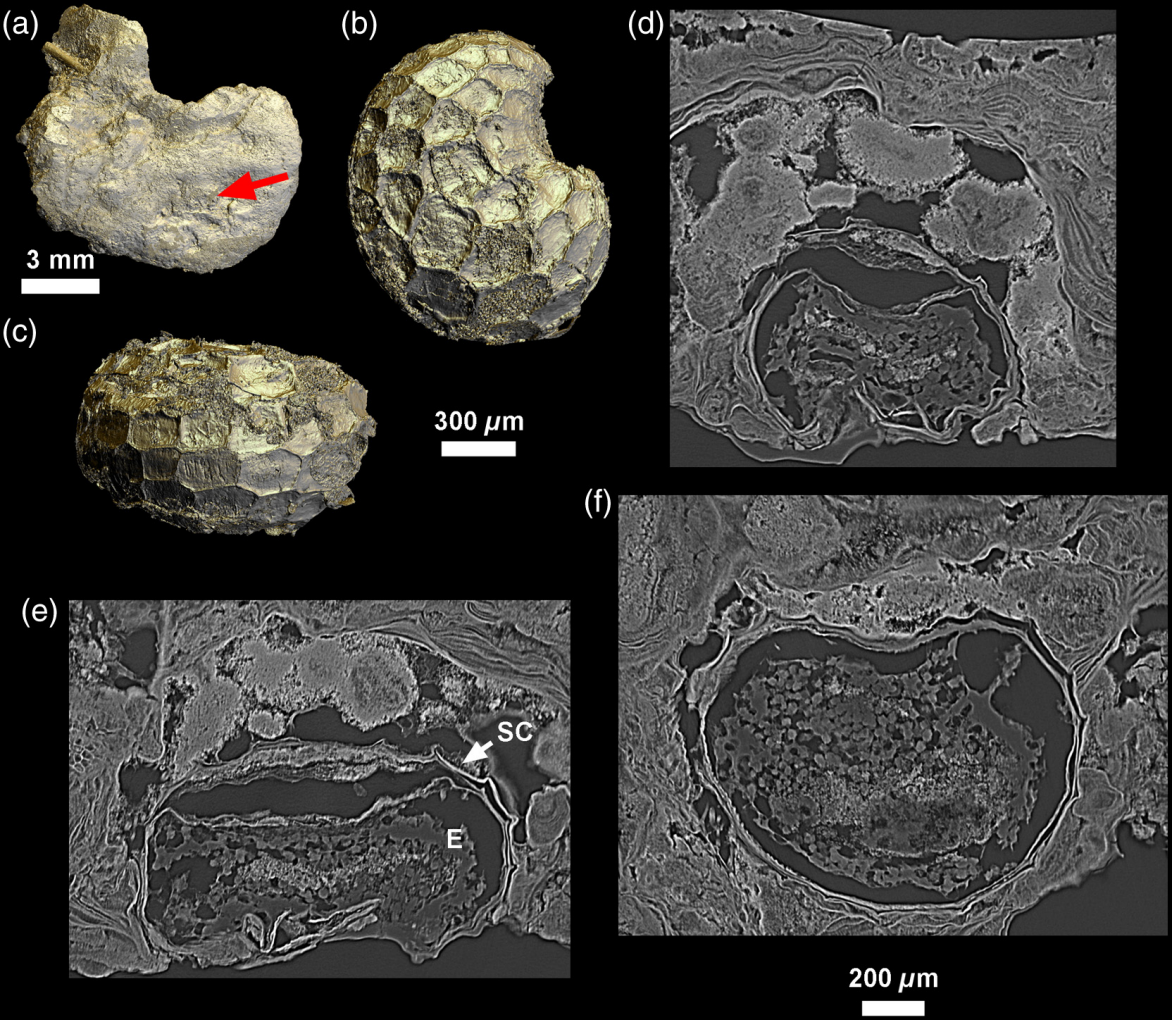
(a) 3D model of the modern specimen CM03 with red arrow showing the location of a poppy seed (*Papaver* cf. *somniferum*); dorsal view in (b), and lateral view in (c) preserved in the calculus. Virtual 2D sections [thickness: 24  μm; (d)–(f)] enable visualization of the detailed inner structure of the seed, preserving its seed coat (SC), and its endosperm (E). All 3D models are computed with Phong renderer and colored lights.

The better quality of the CM03 scan allows for a finer observation of inclusions. Some inclusions were remarkably well-preserved and easy to identify. These included a reniform object, roughly 1225×1005×770  μm, with a thick outer shell, amorphous interior, and a scalloped surface, found in CM03 (Fig. 7). Based on the 3D structure and by comparison with published images (Ref. 44; and see Figs. 3 and 7 in Ref. 45), this was identified as a poppy seed (*Papaver* cf. *somniferum*). The seed coat (SC) is well preserved, and easily identified in 2D and 3D due to its characteristic textured surface. The endosperm (E) is preserved and visible as well (Fig. 7). The seed is entrapped at the very surface of the calculus (toward the oral cavity), and is almost exposed along its entire length.

**Fig. 8:**
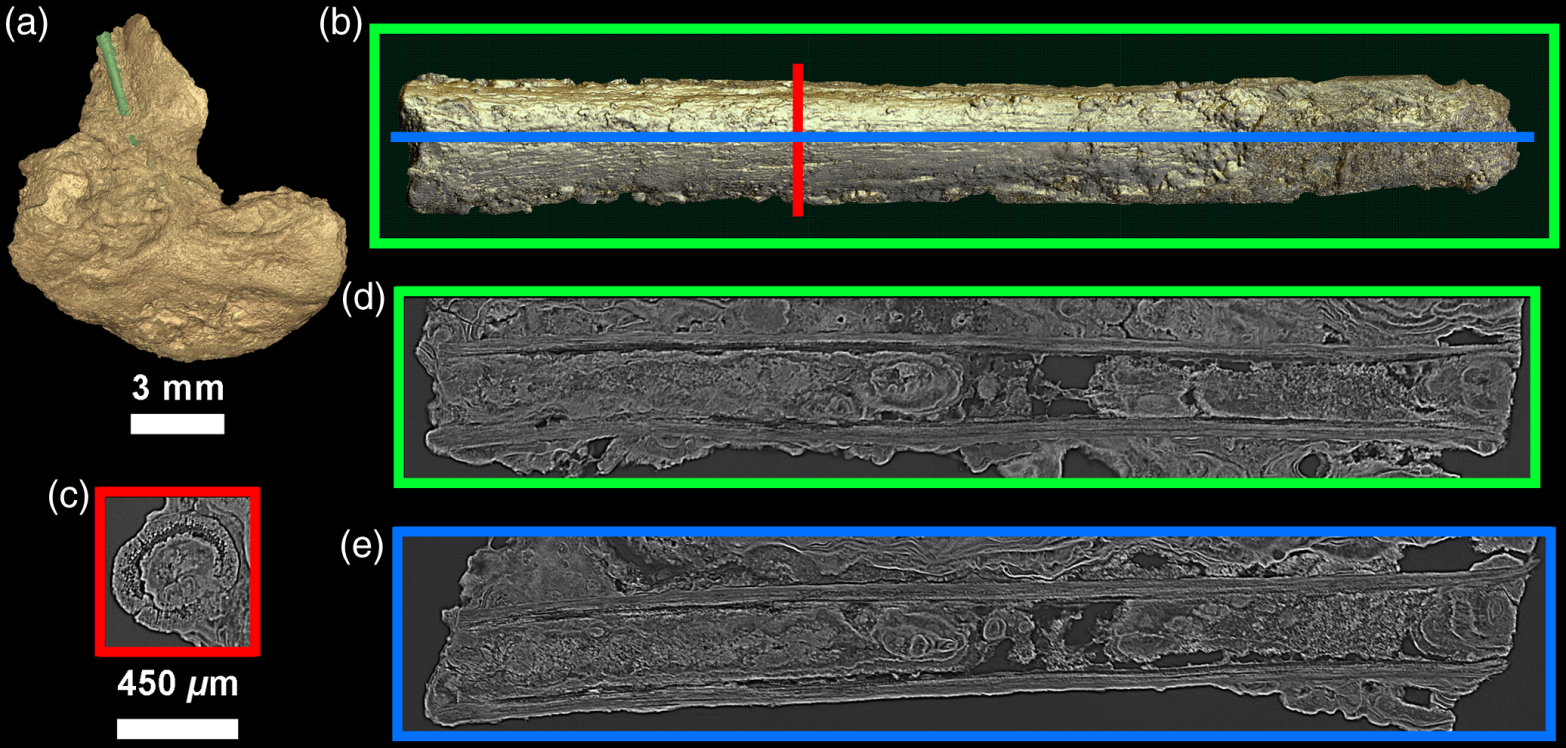
(a) Location of an inclusion likely representing a (b) possible vegetal rod on CM03. The 24-μm-thick virtual 2D sections (d) and (e) reveal a hollow structure with multi-layered walls. Further calculus mineralization events occurred inside this inclusion after it was entrapped in the calculus.

**Fig. 9:**
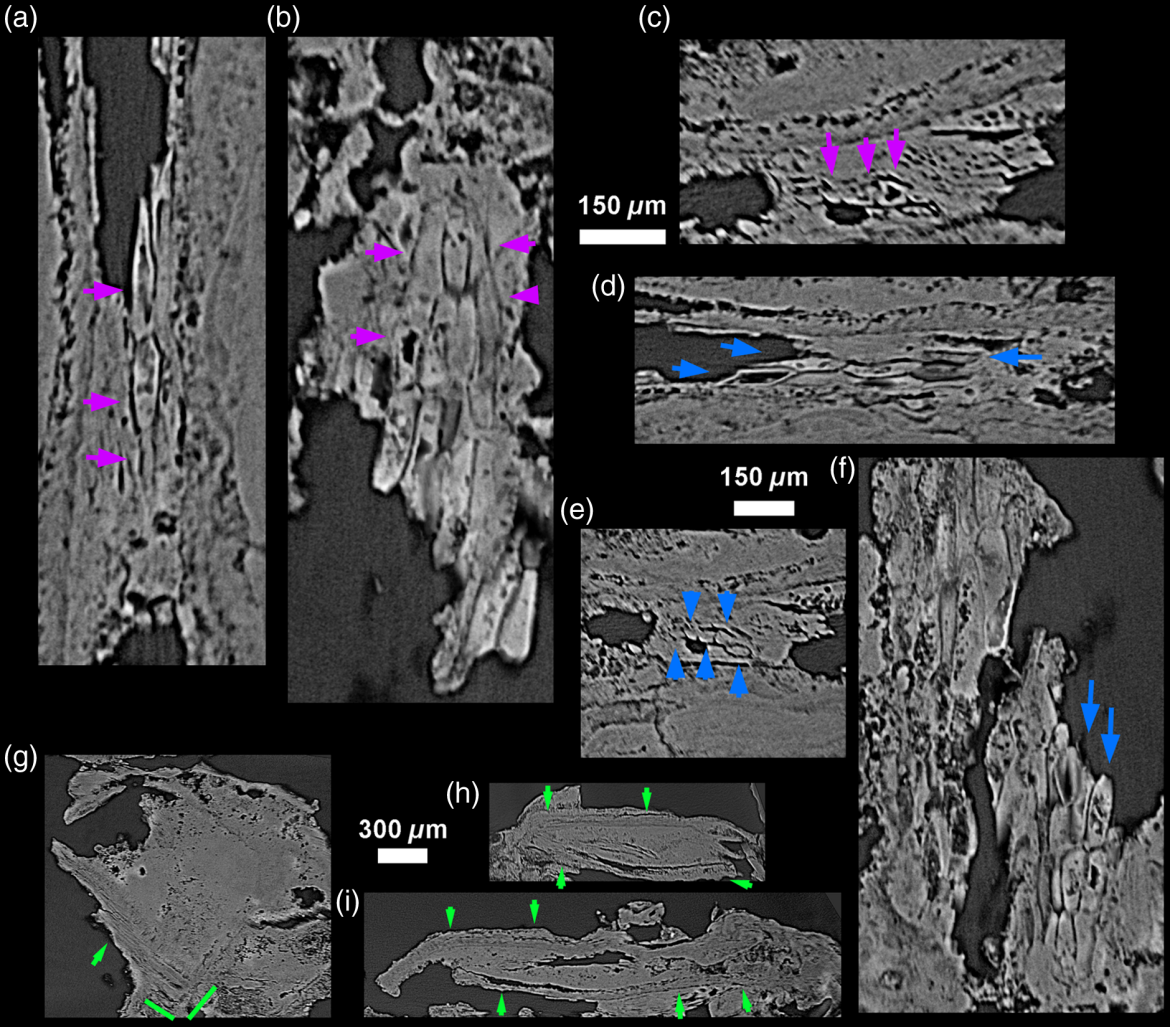
Three inclusions of an uncertain nature from FK01 shown with 12-μm-thick virtual sections in the three planes of space (a)–(c), (d)–(f), and (g)–(i). The first two (a)–(c) and (d)–(f) may represent aligned vegetal cells or fungi, while the third inclusion (g)–(i) looks similar to the rod identified in CM03 (Fig. 8).

Other objects that are clearly inclusions to the calculus were also visible, but we are not yet able to identify their origin. These include a large, 3-mm long tube feature with multi-layer walls, which was partially exposed on the surface of CM03 (Fig. 8), and visible with the naked eye as brown in color. We also noted a smaller rod in FK01, as well as rhomboid cell-like structures, which might be plant cells (Fig. 9).

**Fig. 10:**
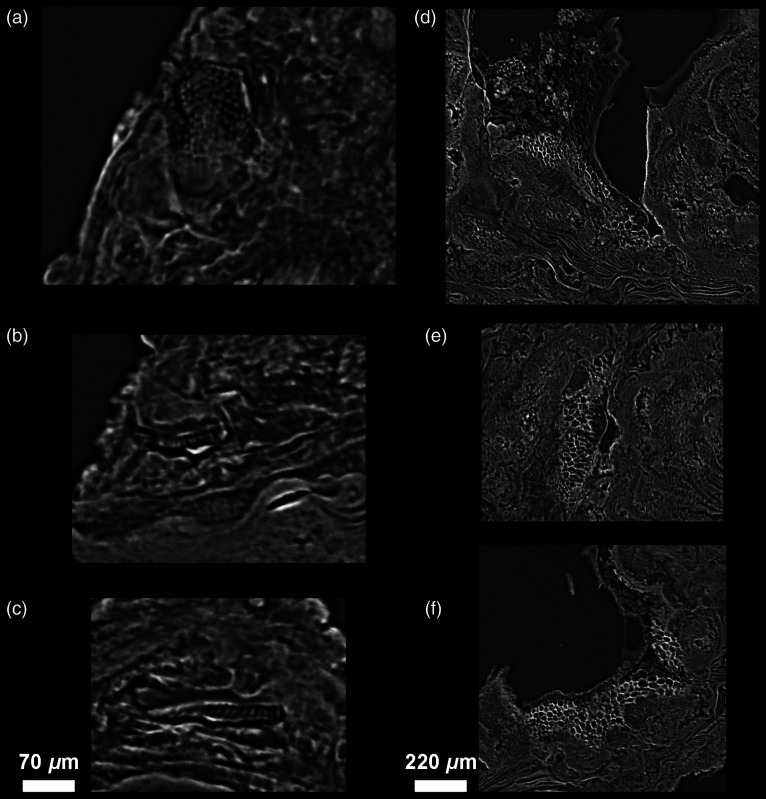
Virtual 2D sections through CM03 showing two instances of inclusions with a honeycomb-like structure. (a)–(c) First inclusion: 18-μm-thick slices; (d)–(f) second inclusion: 24-μm-thick slices.

**Fig. 11:**
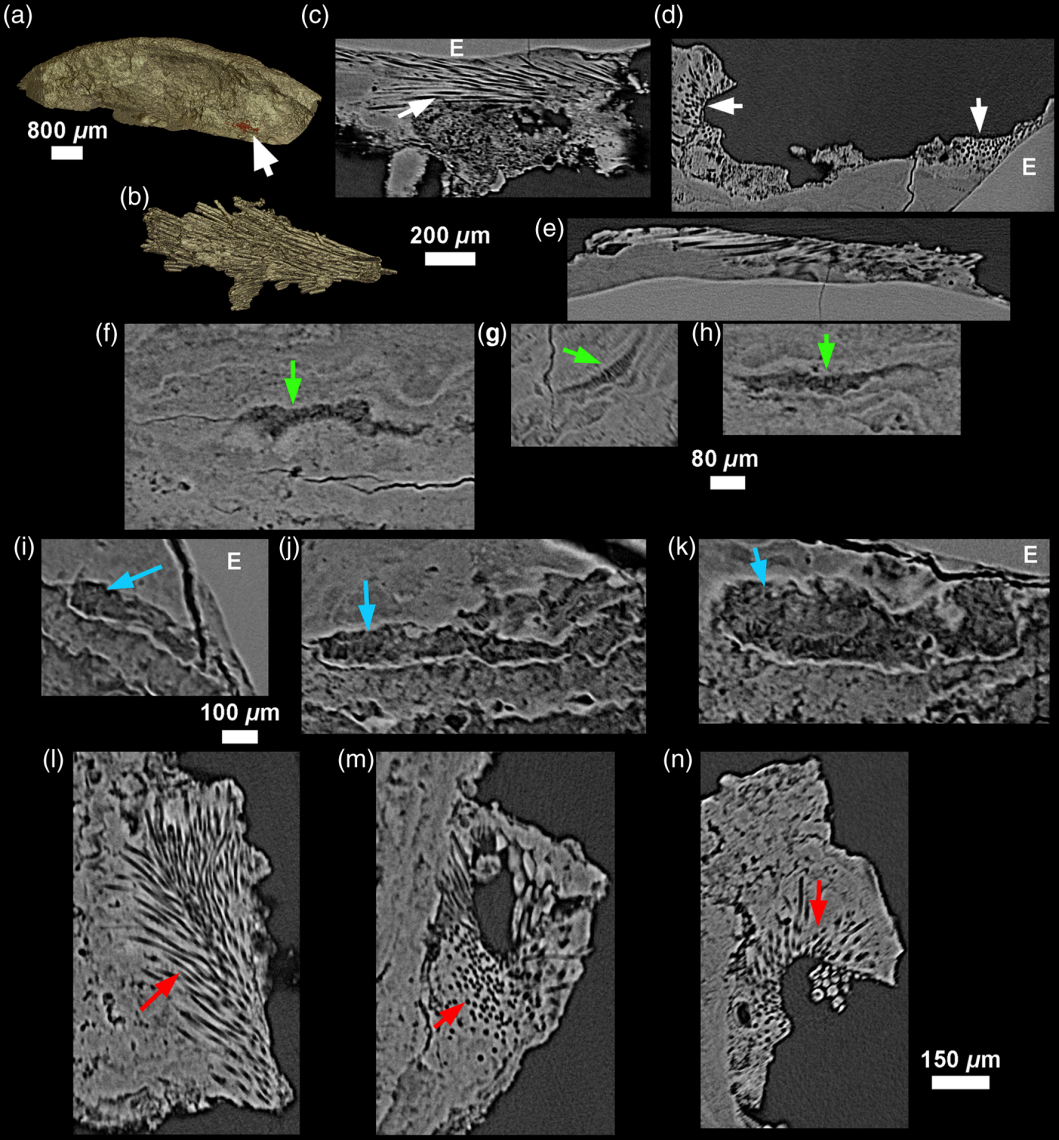
Honeycomb-like structures observed in archeological specimen FK01. The first honeycomb structure is shown in red and indicated by a white arrow on the 3D model of FK01 (a). Virtual 2D sections [8-μm-thick; (c)–(e)] show a regular pattern of empty spaces running parallel to the calculus surface. (b) 3D model depicting the negative imprint of these canals. (f)–(h) The second and (i)–(k) third honeycomb-like structures look slightly different, with less accentuated empty spaces. The third structure (l)–(n) is more similar to (b)–(e). Virtual sections (f)–(n) are 12-μm-thick.

In some cases, discerning the complex structures created by the mineralization of oral microbiome communities from inclusions was challenging. We observed several structures that fall into this category: honeycomb structures that occurred on the edges of voids, and rod-like features that may be pseudomorphs of exudate/transudate from bacteria, or the remnants of the bacteria themselves (Figs. 8–11; see Figs. 3G, 5 and 6 in Ref. 46).^19^

## 5 Discussion and Conclusion

Synchrotron radiation micro-tomography allows unparalleled insights into the inner structure of human dental calculus. This non-destructive imaging technique enables researchers to visualize inclusions *in situ*, without losing their complex preservational contexts. Fragile calcium-based inclusions, which are lost by dissolution in typical extraction methods, can be identified. Many of the potentially informative inclusions found in calculus (e.g., starches, phytoliths, diatoms, pollen, etc.) are ∼5- to 100-μm large, and therefore should be visible with the effective pixel size of 2.55  μm. We did not identify these microremains in this preliminary study but expect that a more thorough examination of our scans would reveal such inclusions.

We show that conventional μCT scans, which are more widely available than PPC-SR-μCT, may reveal some of the inner structure of dental calculus. However, phase contrast (used in PPC-SR-μCT) appears necessary to reach the level of details and spatial resolution that allow the unambiguous identification of the calculus layers and clear visualization of the borders of the inclusions. Conventional μCT may however be easily used as a pre-screening step to select valuable specimens for further investigation at a synchrotron facility.

In contrast to histological and hard tissue sections, which provide layers of 3 to 50  μm with adjoining volume being lost,^47^ PPC-SR-μCT enables us to explore the entire scanned area, or even the entire specimen for an isolated chunk of calculus. Synchrotron radiation μCT provides an infinite degree of freedom regarding slice position, orientation (i.e., rotation and translation), and thickness (Fig. 4). Furthermore, unlike thin sectioning, we are able to create sections in targeted areas of any thickness without the risk of breaking the sample, therefore enhancing reproducibility.

Synchrotron radiation micro-tomography also enables us to easily distinguish, in an isolated calculus fragment, the surface of the specimen that was in contact with the oral environment from that which was in contact with the crown enamel or root surface. The clear imprint of perikymata, periradicular bands and the gingival line on the interior surface suggests that it may be possible to use well-preserved calculus as an insight into the record of dental development (e.g., perikymata counts in preserved areas, perikymata spacing, and enamel hypoplasias) in the absence of the tooth itself.

Shifts in diet, health, or oral pollution could potentially be captured in dental calculus and identified by a change in the nature of the inclusions, in the layers’ thickness or structure (or even in chemical composition, although this is not possible to assess using PPC-SR-μCT). Our results support the possibility of providing a relative chronology of inclusions based on their position within the calculus. Given that calculus develops first on the enamel surface and then builds on itself, inclusions found closer to the enamel will be older than those found near the exterior of the calculus (i.e., toward the oral environment). However, our results clearly demonstrate that the development of calculus does not follow a regular, onion-like pattern. Instead, the layering is irregular, and visually similar to that of stromatolites, with layers appearing in some areas but not in others. The periodicity of these layers is unknown, and likely varies among individuals. Given the highly irregular nature of these layers, as well as frequent presence of voids, methods such as repeated acid etching cannot accurately discern the true depositional order of the inclusions, nor capture the formation process on a fine time scale. It might be possible to use synchrotron images of calculus to follow each layer surface and establish the order of formation, to more closely constrain the relative ages of the inclusions.

Finally, the comparison of the modern and archeological samples provides some insight into the potential taphonomic processes affecting ancient calculus. While some of the differences between these samples are due to changes in the scanning parameters (see Table 1), others such as the increase in voids and cracks in FK01 are likely due to a variety of taphonomic processes, including exposure to sediments, moisture and temperature changes, and weathering. Cracks and voids occur even in modern calculus, and can be much larger even in well-preserved and solid-looking archeological specimens. These open spaces may allow decay to occur inside calculus deposits. We do note, however, that there did not appear to be any in-filling of these cracks, suggesting that larger inclusions were not introduced to the interior of the calculus. However, biomolecules that might be mobilized by water, such as proteins and DNA, may be washed out of or washed into the calculus. Further study of more modern and ancient calculus is needed to determine if taphonomic alteration to calculus is widespread.
